# Reversal of memory and neuropsychiatric symptoms and reduced tau pathology by selenium in 3xTg-AD mice

**DOI:** 10.1038/s41598-018-24741-0

**Published:** 2018-04-24

**Authors:** Ann Van der Jeugd, Arnaldo Parra-Damas, Raquel Baeta-Corral, Carlos M. Soto-Faguás, Tariq Ahmed, Frank M. LaFerla, Lydia Giménez-Llort, Rudi D’Hooge, Carlos A. Saura

**Affiliations:** 1 Laboratory of Biological Psychology, Katholieke Universiteit Leuven, Leuven, 3000 Belgium; 2 Institut de Neurociències, Departament de Bioquímica i Biologia Molecular, Centro de Investigación Biomédica en Red Enfermedades Neurodegenerativas (CIBERNED), Universitat Autònoma de Barcelona, Bellaterra, 08193 Spain; 3 Institut de Neurociències, Departament de Psiquiatria i Medicina Legal, Unitat de Psicologia Mèdica, Universitat Autònoma de Barcelona, Bellaterra, 08193 Spain; 4 Department of Neurobiology and Behavior, Institute for Memory Impairments and Neurological Disorders, University of California, Irvine, 92697 CA USA; 5 Present Address: Neurological Disorders Research Center, Qatar Biomedical Research Institute, Hamad Bin Khalifa University, Doha, Qatar

## Abstract

Accumulation of amyloid-β plaques and tau contribute to the pathogenesis of Alzheimer’s disease (AD), but it is unclear whether targeting tau pathology by antioxidants independently of amyloid-β causes beneficial effects on memory and neuropsychiatric symptoms. Selenium, an essential antioxidant element reduced in the aging brain, prevents development of neuropathology in AD transgenic mice at early disease stages. The therapeutic potential of selenium for ameliorating or reversing neuropsychiatric and cognitive behavioral symptoms at late AD stages is largely unknown. Here, we evaluated the effects of chronic dietary sodium selenate supplementation for 4 months in female 3xTg-AD mice at 12–14 months of age. Chronic sodium selenate treatment efficiently reversed hippocampal-dependent learning and memory impairments, and behavior- and neuropsychiatric-like symptoms in old female 3xTg-AD mice. Selenium significantly decreased the number of aggregated tau-positive neurons and astrogliosis, without globally affecting amyloid plaques, in the hippocampus of 3xTg-AD mice. These results indicate that selenium treatment reverses AD-like memory and neuropsychiatric symptoms by a mechanism involving reduction of aggregated tau and/or reactive astrocytes but not amyloid pathology. These results suggest that sodium selenate could be part of a combined therapeutic approach for the treatment of memory and neuropsychiatric symptoms in advanced AD stages.

## Introduction

Alzheimer’s disease (AD) is characterized by progressive memory decline and emotional and neuropsychiatric symptoms associated with accumulation of amyloid-β (Aβ) plaques and tau-containing neurofibrillary tangles (NFTs)^1^. Cognitive decline correlates better with progression of tau pathology in the hippocampus rather than amyloid plaques in neocortical regions^2^. These classical pathological hallmarks accumulate similarly in the hippocampus and cortex of transgenic 3xTg-AD mice, which develop age-dependent hippocampal-dependent cognitive deficits and neuropsychiatric-like disturbances^3–6^. Targeting Aβ and phosphorylated tau ameliorate and/or reverse memory and synaptic deficits in 3xTg-AD mice^7^, although it is still unclear whether targeting tau independently of Aβ may have therapeutic benefits on cognition and/or emotional symptoms at late AD stages.

Epidemiological studies show imbalance of essential inorganic elements, including selenium (Se), in the brain during aging and AD^8,9^. Plasma levels of Se decline during aging, mild cognitive impairment and AD, and its deficiency is associated with increased risk of developing AD^10,11^. Se regulates critical cellular antioxidant signaling pathways by acting as Se-containing compounds or selenoproteins being beneficial against neurotoxicity and oxidative damage in AD^12^. A number of clinical trials demonstrated preventive or therapeutic effects of Se alone or combinated with antioxidants in multiple human diseases^13^. Randomized clinical trials with Se plus antioxidants are currently in progress in AD but without reported outcome data yet^14,15^.

Sodium selenite and selenate protect hippocampal neurons against Aβ42 toxicity and reduce amyloid burden in APP/PS1 transgenic mice^16–18^. Chronic sodium selenate in drinking water mitigates tau pathology in AD and traumatic brain injury murine models^19,20^. Sodium selenate-treated tau transgenic mice exhibit improved spatial learning and memory and show lower levels of phosphorylated and insoluble tau levels in the hippocampus and amygdala^21,22^. More recently, selenomethionine, supplemented before appearance of pathology, was shown to prevent spatial learning deficits, and reduced total and phosphorylated tau levels by promoting autophagy in mix sex groups of young 3xTg-AD mice^20,23^. Whether Se supplemented as a natural salt reverses emotional and memory symptoms specifically in AD female mice at late stages remain unclear. In this study, we investigated the effects of chronic dietary sodium selenate supplementation on both cognitive and neuropsychiatric-like domains, and studied the pathological mechanisms involved, in old 3xTg-AD mice.

## Results

### Chronic sodium selenate treatment stabilizes idiopathic behaviors in 3xTg-AD mice

**Figure 1:**
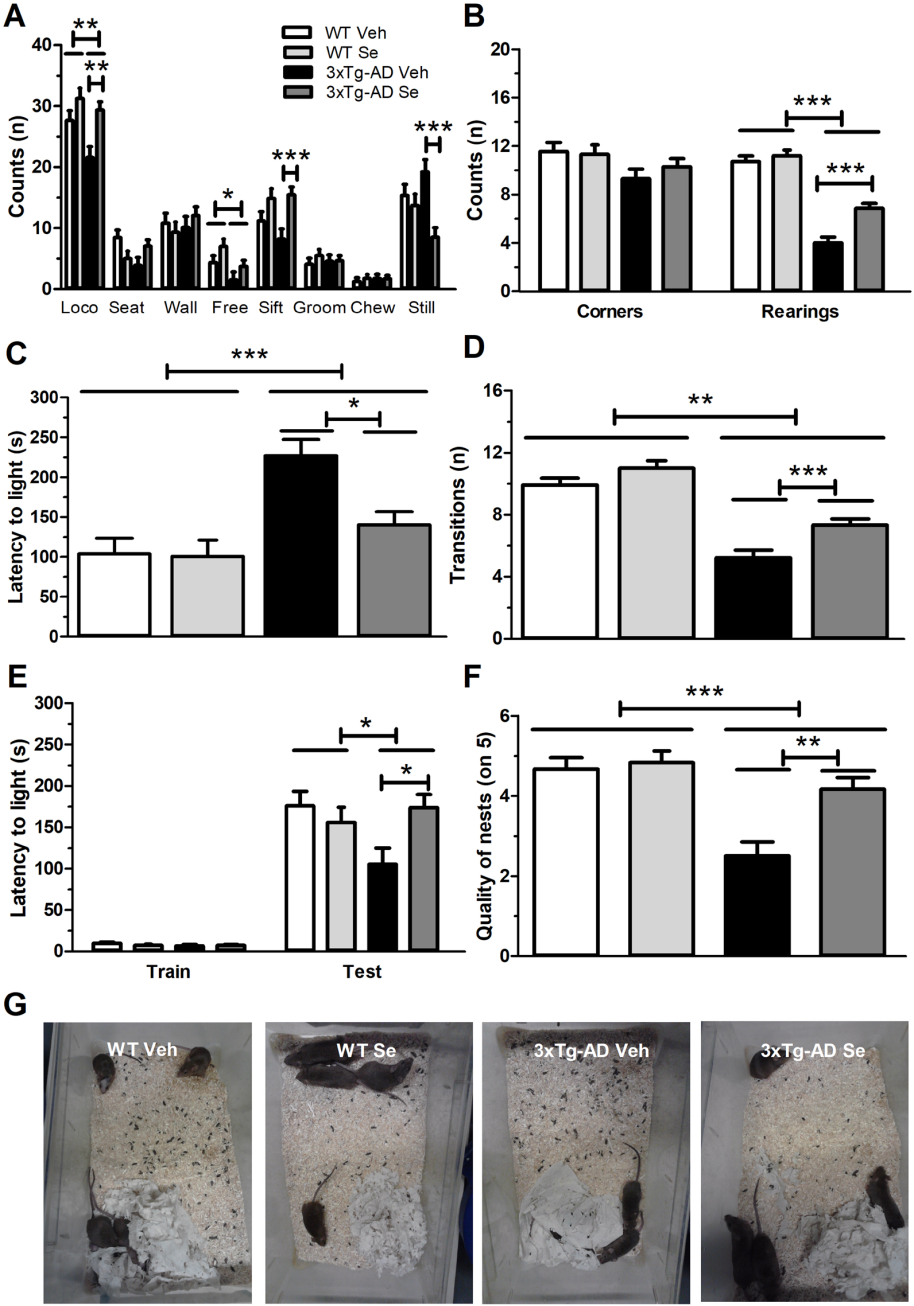
Sodium selenate ameliorates idiopathic and anxiety behaviors in 3xTg-AD mice. (**A**) Ethological assessment of WT and 3xTg-AD mice after chronic vehicle (Veh) or sodium selenate (Se) treatment. Loco: locomotion; seat: rearing seated; wall: rearing against the wall; free: rearing free; sift: sifting; groom: grooming; chew: chewing; still: stillness. (**B**) Evaluation of mice in the corner index task. Counts: number of visited corners or rearings. (**C**,**D**) Assessment of neophobia/anxiety in the dark-light test. 3xTg-AD mice show significantly increased latencies (**C**) and reduced transitions (**D**) towards the light compartment, a behavior reversed by Se treatment. (**E**) Evaluation of fear associative memory in the passive avoidance test. The reduced latencies to enter to the dark compartment were reversed in 3xTg-AD mice after Se treatment. (**F**,**G**) Assessment of well-being and social collaboration in the nest building test. 3xTg-AD mice showed a significant less nest-building ability than WT groups, while Se treatment increases nesting activity in 3xTg-AD mice (**F**) as observed in representative pictures of nests (**G**). Data represent mean ± s.e.m. Number of mice: WT vehicle (n = 11), WT Se (n = 10), 3xTg-AD vehicle (n = 9) and 3xTg-AD Se (n = 15). Statistical analysis was determined by one-way ANOVA followed by Bonferroni *post hoc* test. *P < 0.05, **P < 0.001, ***P < 0.001.

3xTg-AD mice show age- and sex-related differences in brain AD-like pathology and oxidative stress and behavior, which include enhanced amyloid pathology in females^24,25^, and immunological and behavioral alterations in males^26–28^. To evaluate specifically the effects of Se in females, the sex more affected by the disease in humans, we evaluated the effects of 4 months-chronic Se supplementation diet in 12–14 month-old female control (WT) and 3xTg-AD mice, which started treatment at 8 months of age. No significant differences were found in rotarod test between genotype and treatment groups [F (3,44) = 0.512, *P* > 0.05; data not shown]. During ethological assessment, we found no significant differences among groups for rearing seated or against the wall, nor regular or intense grooming and chewing. By contrast, we found significant differences between groups in locomotion [F (3,44) = 5.427, *P* = 0.002], free rearings [F (3,44) = 3.313, *P* = 0.029], sifting [F (3,44) = 5.011, *P* = 0.005] and stillness [F (3,44) = 6.425, *P* = 0.001] (one-way ANOVA; Fig. 1A). Bonferroni test revealed that 3xTg-AD mice showed reduced locomotion (t = 3.926, *P* = 0.002), free rearings (t = 3.103, *P* = 0.021) and sifting (t = 3.472, *P* = 0.007), whereas Se significantly reversed these alterations in locomotion (t = 3.451, *P* = 0.008), sifting (t = 7.24, *P* = 0.001) and stillness (t = 4.197, *P* < 0.001). Corner index measures revealed no group differences on corner visits [F (3,44) = 2.056, *P* > 0.05] but significant rearings differences among groups [F (3,44) = 49.546, *P* < 0.001] (Fig. 1B**)**. 3xTg-AD mice visited slightly fewer corners and performed less rearings compared to WT mice (*P* < 0.001), a phenotype that was significantly ameliorated by Se treatment (rearings: t = 4.276, *P* = 0.001) (Fig. 1B).

When testing anxiety in the dark/light test, 3xTg-AD mice took much longer than all other groups to emerge from the dark compartment [group effect, latency: *F* (3,44) = 7.199, *P* < 0.001; transitions: F (3,44) = 12.29, *P* < 0.001]. *Post-hoc* comparisons revealed differences between vehicle-treated 3xTg-AD and all other groups (all *P* < 0.001), whereas sodium selenate significantly reduced latencies (t = 3.021, *P* = 0.26) and increased transitions (t = 5.724, *P* = 0.001) in 3xTg-AD mice (Fig. 1C,D). In the single-trial passive avoidance test, latencies to enter the dark compartment were not different among groups during the training day (*P* > 0.05). However, significant latencies differences were evident between groups during testing at 24 h [F (3,44) = 3.058, *P* = 0.04], whereas latencies were significantly rescued by Se treatment in 3xTg-AD mice (t = 2.587, *P* = 0.0172; Fig. 1E**)**. When assessing well-being and social collaboration in the nest building test, 3xTg-AD mice showed less nest-building ability than WT groups, whereas Se treatment significantly increased nesting activity in 3xTg-AD mice [F (3,10) = 35.17, *P* < 0.001; Fig. 1F,G].

### Rescue of hippocampus-dependent spatial learning and memory in 3xTg-AD mice by sodium selenate treatment

**Figure 2:**
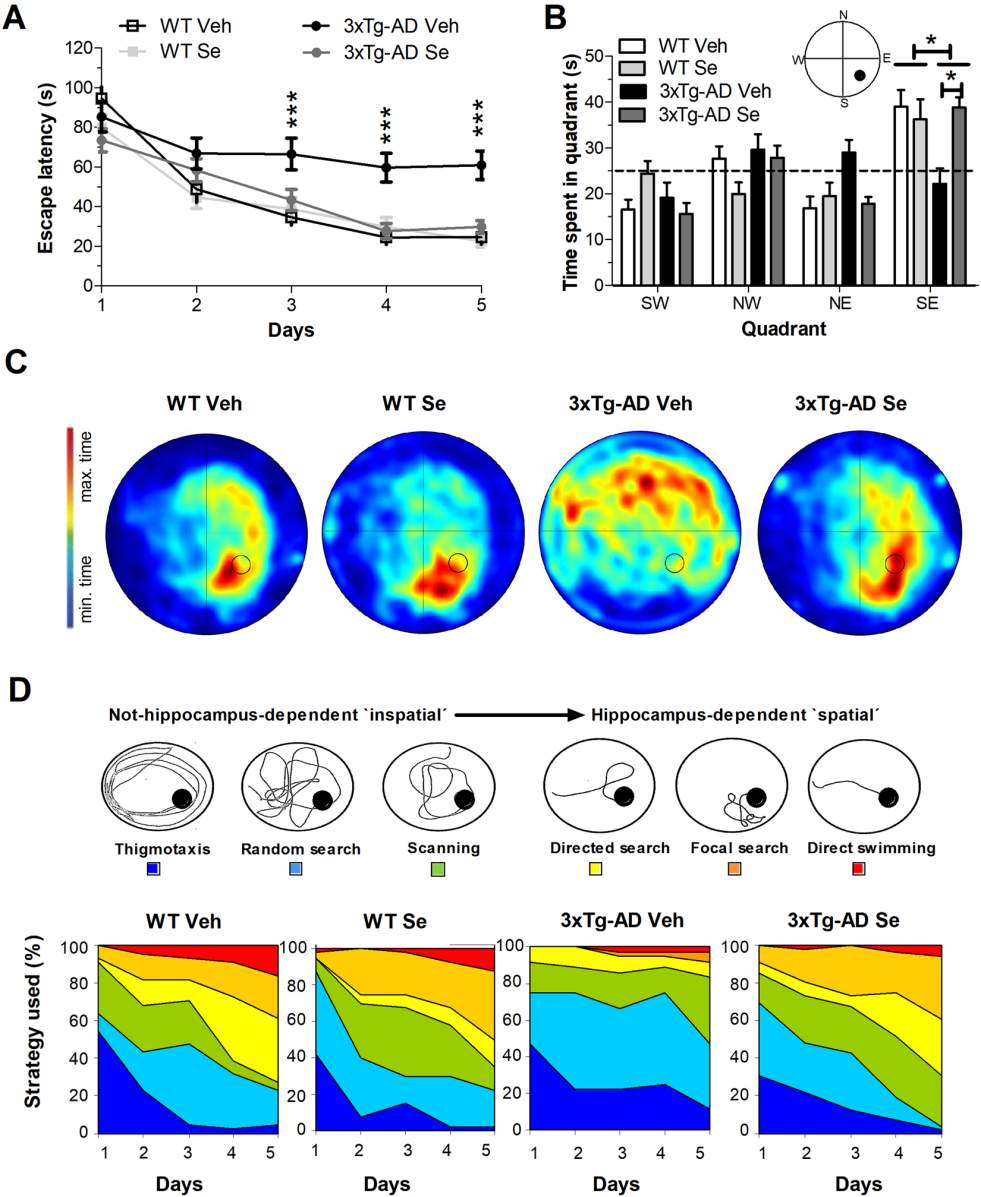
Selenium treatment reverses spatial learning and memory deficits in 3xTg-AD mice. (**A**) Spatial learning curves in the Morris water maze. The spatial training consisted of five-day training in the hidden platform version of the task. The performance of all groups improved significantly during the training days, although 3xTg-AD Veh mice show significantly longer latencies than the rest of groups. (**B**) Spatial reference memory in the MWM after treatment. Results represent the time spent in each quadrant in the post training probe trial. Compared to WT groups, 3xTg-AD Veh mice did not show a preference for the target quadrant (SE), whereas Se treatment enhances target quadrant preference in 3xTg-AD mice. (**C**) Swimming pathlengths during navigation in the probe trial. Heat plots of pathlengths of representative experimental mice during the probe trial. (**D**) Spatial navigation strategies of mice in the MWM. Top: Schematic representations of non-spatial and spatial navigation strategies in the MWM. Bottom: Effect of sodium selenate on non-spatial and spatial learning strategies in the MWM. Data represent mean ± s.e.m. Number of mice: WT vehicle (n = 11), WT Se (n = 10), 3xTg-AD Veh (n = 9) and 3xTg-AD Se (n = 14). Statistical analysis was determined by two-way ANOVA followed by Bonferroni *post hoc* test. *P < 0.05, ***P < 0.0001.

In the Morris water maze (MWM), a spatial memory task that depends on the hippocampus, all experimental groups improved significantly during the training days but with significant learning differences between the groups [Two-way RM ANOVA; Escape latencies: group effect, F (3,40) = 7.74, *P* < 0.001; day effect, F (4,160) = 43.514, *P* < 0.001; Pathlengths: group effect, F (3,40) = 15.66, *P* < 0.001; day effect: F (4,160) = 33.5752, *P* < 0.001; Fig. 2A]. Post hoc comparisons revealed that untreated 3xTg-AD mice displayed longer escape latencies in comparison to other groups at days 3 to 5 (all *P* < 0.001). Swimming velocities were not different between the groups [F (3,40) = 2.305, *P* > 0.05], but all mice became faster and better swimmers during the days (F (4,160) = 3.887, *P* < 0.01). In the probe trial, both WT groups displayed a clear preference searching in the target quadrant (WT Veh: 39% time; WT Se: 36%) compared with 3xTg-AD mice (22%) (Fig. 2B,C). After Se treatment, 3xTg-AD mice spent significantly more time in the target quadrant (39%) indicating rescue of reference memory [F (3,43) = 5.121, *P* = 0.004]. Indeed, heat plots reveals that control groups and Se-treated 3xTg-AD mice mostly searched close to the designated platform position, whereas vehicle 3xTg-AD mice seemed to be circling more aimlessly (Fig. 2C).

During acquisition training, different spatial search strategies can be used (Fig. 2D). When counting the number of trials categorized in three main search strategies, we observed that both vehicle and Se-supplemented WT mice increasingly applied spatial strategies to locate the hidden platform (Fig. 2D). On day 5, vehicle- and Se-supplemented WT mice almost exclusive used hippocampal-dependent spatial strategies (72% and 65%, respectively), compared to vehicle 3xTg-AD mice (17%), which tended to use scanning and random strategies (36%). Notably, the use of spatial strategies increased after Se treatment in 3xTg-AD mice (69%).

### Sodium selenate does not affect accumulation of amyloid plaques nor intraneuronal Aβ

**Figure 3:**
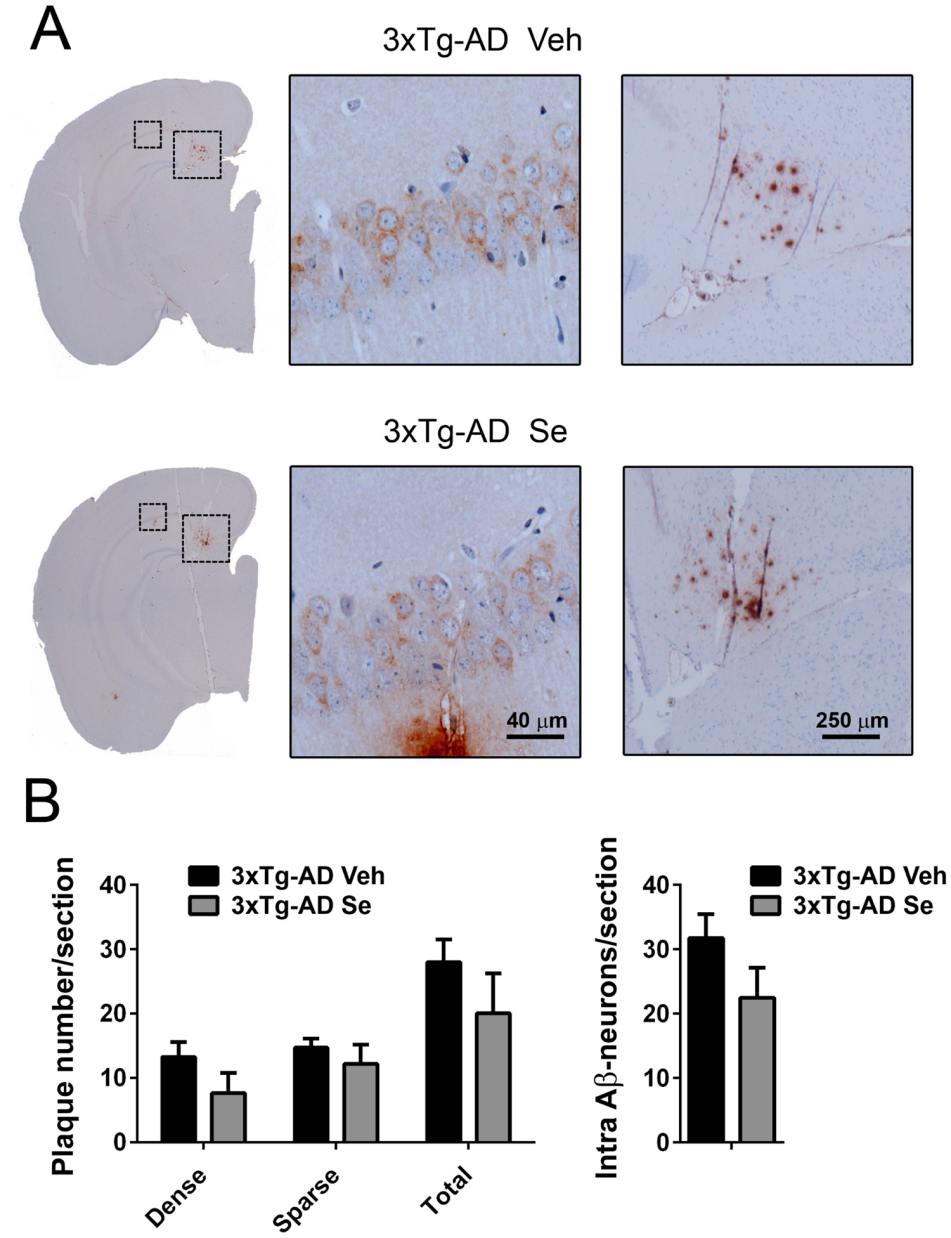
Amyloid pathology is not affected by selenium treatment in 3xTg-AD mice. (**A**) Cerebral amyloid pathology in 3xTg-AD mice at 13–14 months of age. Coronal brain sections of vehicle- (Veh) and Se-treated 3xTg-AD mice were stained with an Aβ antibody (6E10) to reveal the presence of intraneuronal Aβ (middle image) and amyloid plaques (right image) in the hippocampus. The middle and right images are magnified images of the left dashed areas (insets) corresponding to CA1 and subiculum subregions. Scale bars = 40 μm and 250 μm. (**B**) Quantification of amyloid pathology in the treated groups. Results include number of dense, sparse or total Aβ plaques and intraneuronal Aβ-positive neurons per section, for brain regions shown in (**A**) Data represent mean number ± s.e.m. n = 4–5 sections/mouse; n = 6–7 mice/group.

To determine whether Se could be beneficial in cognition by affecting AD-related pathological hallmarks, we next performed Aβ histopathological analyses in the above treated groups. 3xTg-AD mice accumulate abundant intracellular Aβ and amyloid plaques in the hippocampus, especially in the subiculum, and only sporadically in the neocortex both in vehicle- and Se-treated conditions (Fig. 3A). Quantitative analysis revealed no significant differences in the number of dense, sparse or total amyloid plaques in the hippocampus between vehicle- and Se-treated 3xTg-AD mice (*P* > 0.05; Fig. 3B). Since intraneuronal Aβ is one of the earliest neuropathological hallmarks of 3xTg-AD mice that coincides with memory deficits^4^, we next quantified Aβ-containing neurons in the hippocampus. Quantitative analyses showed no significant changes in the number of Aβ-positive CA1 pyramidal neurons between vehicle- and Se-treated 3xTg-AD mice (*P* > 0.05; Fig. 3A,B).

### Sodium selenate reduces accumulation of aggregated tau in 3xTg-AD mice

**Figure 4:**
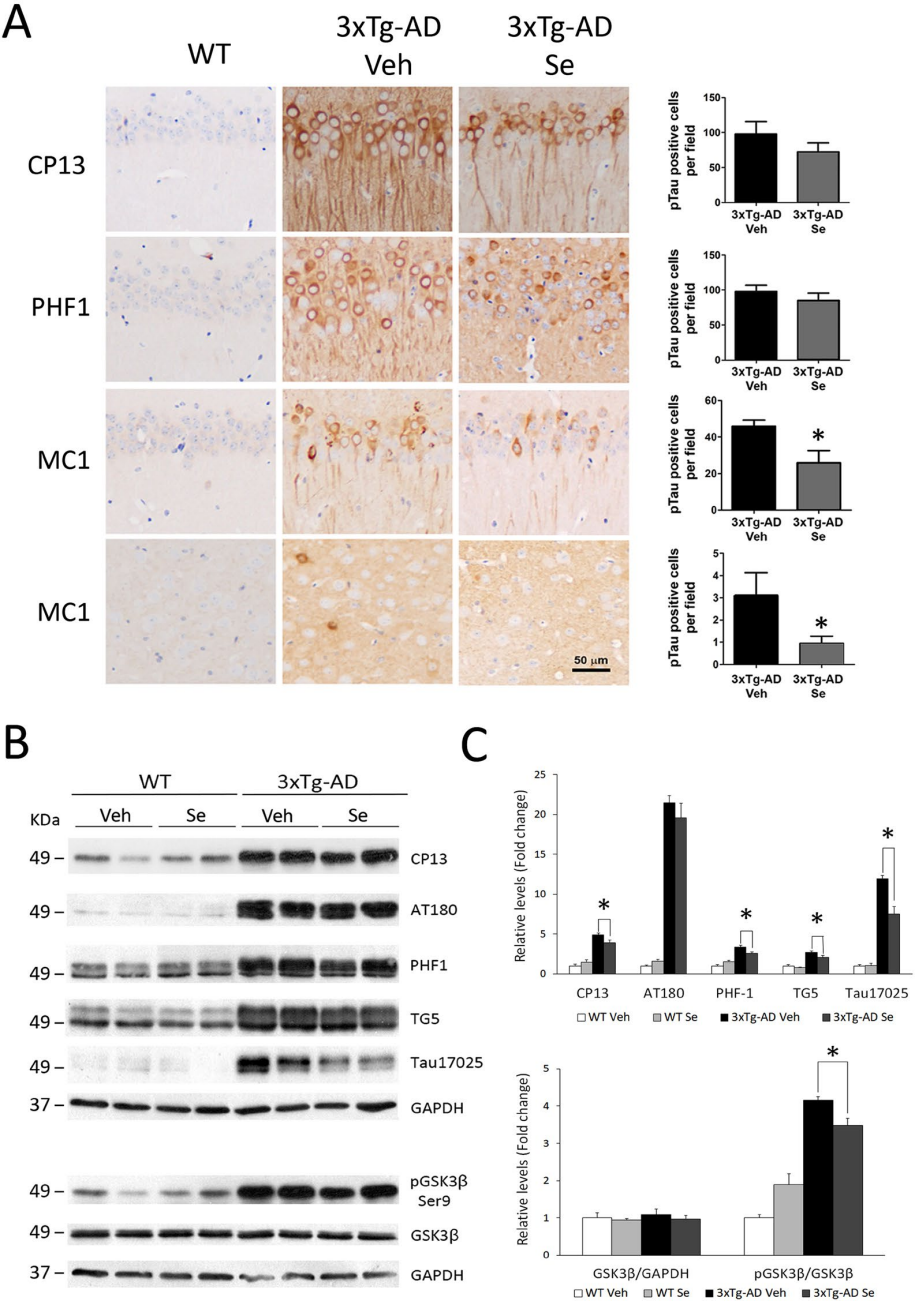
Tau pathology is reduced by selenium treatment in 3xTg-AD mice. (**A**) Cerebral tau pathology is present in the brain of 3xTg-AD at 13–14 months of age. Images show coronal brain sections of WT control (left) and vehicle- (middle) or Se (right)-treated 3xTg-AD mice stained with antibodies against phosphorylated tau (CP13: Ser202; PHF1: Ser396/Thr404) and conformational aggregated tau (MC1) found in AD. Upper three rows correspond to CA1 hippocampus and the bottom row images correspond to the temporal auditory cortex. Scale bar = 50 μm. Right: quantitative imaging analysis of phosphorylated or aggregated tau-positive neurons in vehicle- and Se-treated 3xTg-AD mice. Data represent number of tau-positive neurons per selected region ± s.e.m. n = 4–5 sections/mouse; n = 6–7 mice/group. (**B**,**C)** Biochemical analysis of tau and GSK3β in the hippocampus of WT and 3xTg-AD mice. Western blot images (**B**) and quantitative analysis (**C**) of phosphorylated tau (CP13, AT180 and PHF1), total tau (TG5 and 17025) and total and phosphorylated (Ser9) GSK3β. Original immunobloting scans and parts of the blots used for the figures are shown in Supplemental Information. Data represent relative levels of total and phosphorylated proteins as fold change ± s.e.m. n = 4–5 mice/group. Statistical analysis was determined by t-test (**A**) or two-way ANOVA followed by Bonferroni *post hoc* test (**C**). *P < 0.05, **P < 0.001 compared to 3xTg-AD Veh group.

**Figure 5:**
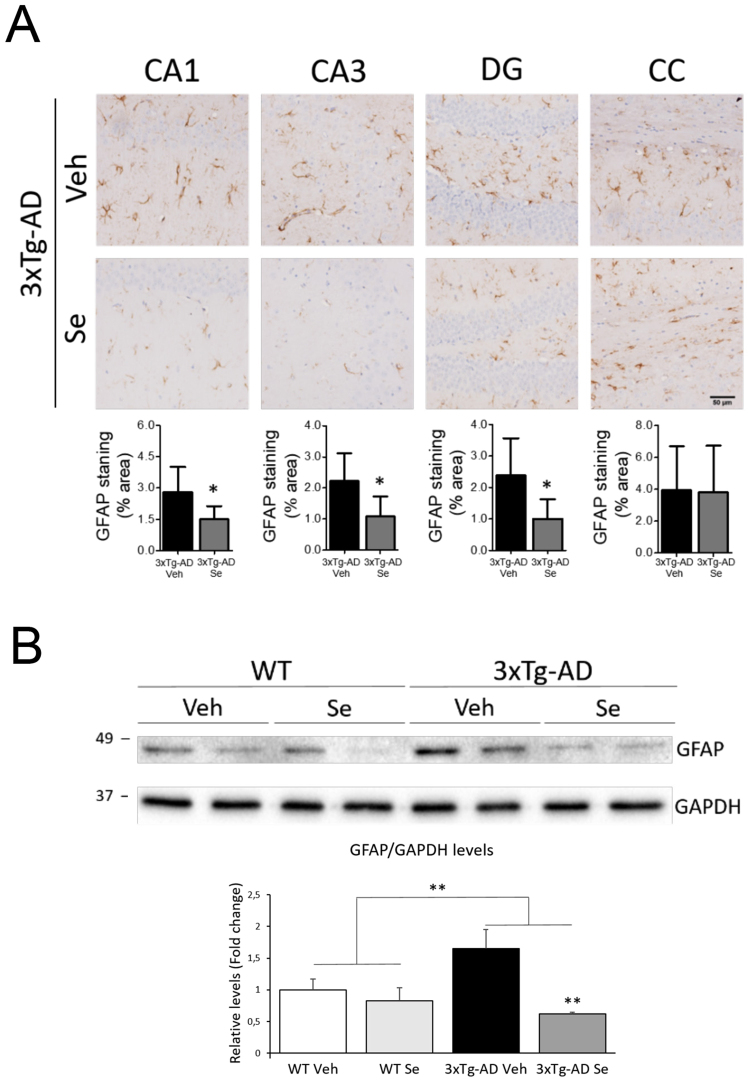
Chronic selenium treatment reduces astrogliosis in the hippocampus of 3xTg-AD mice. (**A**) Immunohistological analysis of astrocytes in 3xTg-AD mice. Coronal brain sections stained with GFAP antibody to detect reactive astrocytes in the hippocampus of 14 month-old 3xTg-AD mice treated with vehicle (Veh) or Se for four months. Images show reduced GFAP staining in the CA1, CA3 and DG regions but not in the corpus callosum (CC) of 3xTg-AD mice treated chronically with Se (bottom) compared with vehicle (top). Scale bar = 50 μm. Data represent percentage of area stained with GFAP ± s.e.m. n = 4–5 mice/group. Statistical analysis was determined by t-test *P < 0.05 compared to 3xTg-AD Veh group. (**B**) Biochemical analysis of GFAP in the hippocampus of treated mice. Western blot images and quantification of GFAP levels in the hippocampus of WT and 3x-Tg-AD mice. Se reduces GFAP levels in the hippocampus of 3xTg-AD mice. Original immunobloting scans and parts of the blots used for the figures are shown in Supplemental Information. Data represent fold relative levels of GFAP ± s.e.m. n = 4–6 mice/group. Statistical analysis was determined by two-way ANOVA followed by Bonferroni *post hoc* test. **P < 0.001.

3xTg-AD mice develop age-dependent tau pathology starting in the basolateral amygdala at 6–9 months and cortex and hippocampus at 12 months of age^3,6^. Staining of 3xTg-AD brains with anti-phosphorylated (CP13, PHF1) and conformational (MC1) tau antibodies show abundant tau accumulation in soma and axons of neurons in the hippocampus (CA1, CA2, CA3, DG), amygdala and cortex (parietal, auditory and entorrhinal cortices) (Fig. 4A). Quantitative analyses revealed no major changes in the number of phosphorylated tau-positive neurons in all regions analyzed in 3xTg-AD mice after Se treatment (*P* > 0.05; Fig. 4A, Table 1). The number of MC1-positive neurons was, however, significantly reduced in CA1 hippocampus and auditory cortex in 3xTg-AD mice after Se treatment (*P* < 0.05; Fig. 4A, Table 1). Western blot analysis showed a significant increase of total (TG5 and 17025 antibodies) and phosphorylated tau at Ser202 (CP13), Thr231 (AT180), Ser396/404 (PHF1) residues in hippocampal lysates of vehicle-treated 3xTg-AD mice compared with WT groups (*P* < 0.05; Fig. 4B). In 3xTg-AD mice, Se treatment slightly decreases phosphorylated tau levels but even more significantly reduced total tau levels, as analyzed with two distinct antibodies (TG5 and 17025) (*P* < 0.05; Fig. 4B,C). Surprisingly, biochemical analysis showed a significant increase of phosphorylated (Ser9)/total GSK3β ratio in the 3xTg-AD groups (*P* < 0.001), which inversely reflects its enzymatic activity (Fig. 4B,C). Finally, immunostaining revealed that 3xTg-AD treated with Se show a global significant reduction of GFAP staining in CA1, CA3 and DG regions (*P* < 0.05) but not in corpus callosum (Fig. 5A). Biochemical analysis confirmed a significant increase of GFAP protein levels in hippocampal lysates of 3xTg-AD mice, which were significantly reduced after Se treatment (*P* < 0.001; Fig. 5B). Together, these results indicate that chronic sodium selenate treatment reduces accumulation of aggregated tau and astrogliosis without globally affecting phosphorylated tau in the hippocampus of 3xTg-AD mice.

**Table 1:** Quantitative analysis of tau pathology in the brain of 3xTg-AD mice after vehicle and selenium treatment.

Brain Region	CP13 (pSer202)		PHF1 (pSer396/404)		MC1 (Tau conf.)	
	Veh	Se	Veh	Se	Veh	Se
CA1	97.7 ± 17.9	72.3 ± 13.1	97.8 ± 9.1	85.2 ± 10.3	45.9 ± 3.3	26 ± 6.6^*^
CA2	68.7 ± 10.4	49 ± 10.3	87.4 ± 20.5	68 ± 16	16.5 ± 7.1	10.5 ± 3.2
CA3	70 ± 14.8	70.8 ± 15.3	79.3 ± 20.5	94.8 ± 26.4	5.5 ± 1.7	5.8 ± 2.1
DG	44.6 ± 8.8	49.9 ± 8.7	43.2 ± 8.1	58.4 ± 13.3	1.8 ± 0.7	1.1 ± 0.3
PCx	124.4 ± 22.9	127.6 ± 16.7	310.1 ± 42.5	297.5 ± 38	0.3 ± 0.1	0.5 ± 0.3
ACx	159.4 ± 15	143.8 ± 15.5	274.1 ± 21	231.8 ± 24.6	3.1 ± 1	0.9 ± 0.3^*▲^
ECx	152.1 ± 16.3	133.8 ± 24.5	205.9 ± 25.8	224 ± 72.5	ND	ND
BLA	135.8 ± 11.4	110.3 ± 26.9	214.5 ± 16.2	200 ± 23	14.3 ± 2	11.3 ± 3.7

Data indicate the number of phosphorylated (CP13: Ser 202; PHF1: Ser 396/404) or aggregated (MC1) tau-positive neurons/field of different brain sections (n = 4–5) per mouse (n = 6–7 mice/group). Abbreviations: CA1, CA2, CA3: Hippocampal Cornu Ammonis 1/2/3 regions, DG: dentate gyrus, PCx: Parietal cortex; ACx: Auditory cortex, ECx: Entorhinal cortex, BLA: Basolateral amygdala. ND: not determined. ^▲^Temporal auditory cortex. *P < 0.05 vs Vehicle.

## Discussion

There is growing evidence that besides genetic and non-genetic risk factors dementia is influenced by a number of environmental factors. A recent review of sixty studies reveals only a limited number of environmental risk factors for dementia, including selenium^9^, an inorganic element whose deficiency is associated with increased risk of developing AD^10,29,30^ (see^11^ for review). Importantly, our study shows that chronic treatment with sodium selenate, a common natural source of Se, efficiently reverses neuropsychiatric and cognitive symptoms in old 3xTg-AD female mice with advanced amyloid and tau pathologies. Importantly, memory improvement is associated with reduced total/aggregated tau and astrogliosis but unchanged Aβ pathology in the hippocampus. These results suggest for the first time that reducing pathological tau with Se may be useful for the treatment of memory and neuropsychiatric symptoms in AD.

Our results indicate that chronic Se treatment efficiently ameliorates neuropsychiatric-like symptoms of dementia, whose effects contribute significantly to the clinical profile in mild cognitive impairment and AD^31,32^. As previously reported^5^, 3xTg-AD mice display less exploratory behavior, as revealed by reduced locomotor activity, free rearings, sifting behaviors and visiting fewer corners and increased stillness. This behavioral phenotype could reflect a subtle change in idiopathic behaviors, since walling, grooming and chewing are not altered. Interestingly, Se treatment has positive effects on fine motoric properties, and anxiety as evaluated by corner visit behaviors, and does not affect gross motor functions nor working memory. In addition, Se improves nest building, an ethological behavior involving high executive function, and therefore useful to assess progressive loss of executive function seen in AD^33^. Besides, nesting behavior is sensitive to apathy and neophobia^34^, two common neuropsychiatric symptoms in individuals with AD^31,35^. Se also reduces neophobic, anxiety and fear-related behaviors in 3xTg-AD mice, a result consistent with increased locomotor and rearing activities. This result is relevant since emotional symptoms, including anxiety and fear, are early clinical features of mild cognitive impairment and AD^36^, although the cellular mechanisms involved are largely unknown. Importantly, accumulation of intraneuronal Aβ and phosphorylated tau in the basolateral amygdala seems to contribute to these symptoms^6^, whereas Se treatment causes a slight non-significant reduction in tau pathology in this region in 3xTg-AD mice (Table 1). While tau pathology in the amygdala could be involved in the emotional disturbances observed in AD, understanding the molecular mechanisms by which Se ameliorates AD-like neuropsychiatric symptoms will be relevant to treat the psychiatric and behavioral symptoms of dementia.

AD mouse models develop memory deficits associated with disrupted synaptic function in the hippocampus. We observed impaired spatial learning and memory in old 3xTg-AD mice likely due to use of less directed search navigation strategies^4,6,37^. This is in concordance with studies showing that hippocampus-impaired animals use spatial search strategies less frequently than controls^38–40^. Se treatment increases the use of direct spatial strategies (e.g., direct swimming, focal search…) resulting likely in memory improvement in 3xTg-AD mice. Indeed, sodium selenate and selenomethionine increase spatial learning in the water maze in young AD transgenic mice^20,22,41^, and a multi-nutrient diet containing Se alleviates cognitive deficits in APP/PS1 mice by increasing the use of navigation search strategies^39^. This effect was mediated by reduction of Aβ and amyloid plaques in young APP/PS1 mice, while it was independent of brain amyloid pathology in old mice^39,42^. Importantly, our results indicate that sodium selenate does not affect the number of amyloid plaques nor intracellular Aβ-containing neurons in old 3xTg-AD mice. Differences in the inorganic (e.g. sodium selenate) and organic (e.g. selenomethionine) source and metabolic fate of the distinct Se compounds may result in different and/or additional therapeutic properties. Nonetheless, the hippocampal-dependent memory recovery by Se is reinforced by rescue of associative memory in the passive avoidance task, a similar result than that found in tau transgenic mice^21^. Although the pathological mechanisms underlying Se-mediated hippocampal-dependent memory recovery are still unclear, the fact that Se reduces total and aggregated tau in the hippocampus strongly suggests a tau-mediated mechanism. In 3xTg-AD mice, tau pathology begins in the basolateral amygdala and then extends to the hippocampus and cortex, whereas hippocampal synaptic transmission and plasticity deficits are evident at 6 months of age or even earlier^3,6,43^. Besides that treatment was extended during the progression of pathological and behavioral features (i.e., from 8 to 12–14 months of age), Se reduced aggregated tau-positive neurons in CA1 hippocampus and temporal auditory cortex in 3xTg-AD mice. Notably, decreasing endogenous tau or reducing tau pathology independently of Aβ ameliorate memory deficits in AD mice^44,45^. By contrast, phosphorylated tau was not globally affected by Se treatment, a result in accordance with unchanged inactive phosphorylated GSK3β levels. Recent investigations show, however, that Se mitigates tau hyperphosphorylation and aggregation by modulating Akt/GSK3β and/or PP2A activities and autophagy pathways^20–23^. Our results reinforce the view that tau pathology contributes to AD-like neuropsychiatric and memory deficits, and that reducing aggregated tau without affecting amyloid pathology is beneficial in 3xTg-AD mice.

The exact mechanism(s) mediating functional recovery of memory neural circuits by Se is unclear. Age-dependent mitochondrial dysfunction and reduced metal homeostasis and antioxidant defense directly affect synaptic function leading to memory loss, whereas synaptic failure is an early pathological feature that plays an important role in cognitive impairment in AD^46–48^. Whether sodium selenate reverts hippocampal-dependent memory deficits by affecting synaptic function remains unclear. One possibility is that seleno-derivatives, acting as antioxidants may affect synaptic transmission and/or plasticity^49^. Indeed, 3xTg-AD mice develop neuronal oxidative stress caused by reduced glutathione and increased reactive oxygen species^50^, whereas selenomethionine increases glutathione levels in these mice^20^. Moreover, disrupted oxidative stress and energy metabolism affect the production and accumulation of Aβ and tau pathologies, which can exacerbate mitochondrial and synapse dysfunction^46^. It is tentative to speculate that Se improves synaptic function leading to memory and psychiatric recovery by reducing directly or indirectly oxidant and tau toxic species. Since tau is also involved in synaptic pathology in AD^51^, investigation of synaptic mechanisms regulated by Se may be important for developing future therapeutic strategies.

In summary, chronic Se supplementation efficiently ameliorates neuropsychiatric-like and memory symptoms and reduces tau aggregation at advanced AD pathological stages. Considering that Se and antioxidant compounds progressively decrease in brain and plasma in mild cognitive impairment and AD patients^52–54^, our results may have important diagnostic and clinical implications. In this context, a signature of at least six essential inorganic elements in serum that includes Se was proposed recently to monitor AD progression^55^. Furthermore, a sodium selenate diet is safe, tolerated and improves neuroimaging measures in mild-moderate AD patients^14^. Nutritional supplementation with Se may represent a promising therapeutic strategy to ameliorate and/or reverse neuropsychiatric and cognitive symptoms in dementia.

## Materials and Methods

### Experimental design and transgenic mice

Triple-transgenic AD mice (3xTg-AD; C57BL/6 × 129 background) harboring the human PS1 (M146V), APPSwe (K670N/M671L) and tau (P301L) transgenes and wild-type (WT) mice^3^, were bred at the Universitat Autònoma de Barcelona (UAB). All mice were females (3xTg-AD = 24 and WT = 21) housed in groups of 3–6 per cage in standard laboratory conditions, including a 12 h light/dark cycle and *ad libitum* access to water and food pellets (Brand: ssniff® R/M-H, containing 0.3 mg Se/Kg; ssniff Spezialdiäten GmbH, Germany). We treated 8 month-old mice with vehicle (3xTg-AD, n = 9; WT, n = 11) or 12 μg/ml sodium selenate (Se; Sigma-Aldrich/Merck, Darmstadt, Germany) (3xTg-AD, n = 15, one died during testing; WT, n = 10) added to the drinking water ad libitum, and continued throughout behavioral testing. Chronic treatment with Se-Met (6 μg/ml) in drinking water was shown previously to elevate Se in the cortex and hippocampus (1.2–1.5 mg/kg) indicating incorporation of Se in the brain^20^. The mice were approximately 12 months old at behavioral testing, and 13–14 months old at physiological, biochemical and pathological analyses.

### Behavioral experiments

Behavioral testing successively included the following tests (in this order): rotarod, ethogram, nesting behavior, corner index, dark/light box, Morris water maze and passive avoidance. Motor coordination and equilibrium were tested on an accelerating rotarod (MED Associates Inc., St. Albans, Vermont, USA). Mice were first trained on a constant speed (4 rpm, 2 min), before starting with four test trials (inter-trial interval, 10 min). During these trials, mice had to balance on the rotating rod that accelerated from 4 to 40 rpm within 5 min. The latency until the mice fell from the rotating rod was recorded, up to a maximum of 5 min^56,57^. Ethological assessments were carried out using a rapid time-sampling behavioral checklist technique, as described previously^58^. Each mouse was observed individually for 5 s periods at 1 min intervals over 15 consecutive min, using an ethologically based behavioral checklist (occurring alone or in any combination) that included: sniffing, locomotion, rearing seated, rearing to wall, rearing free, sifting, grooming, intense grooming, chewing and stillness. This cycle of assessment was repeated twice over an initial exploratory period of 60 min, total counts for each behavior were analysed. Nesting behavior was evaluated by introducing a piece of paper tissue inside the home cage. The presence and quality of the nest were rated two days later on a 1 to 5 point scale^34^: 1 not noticeably touched, 2 partially torn up, 3 mostly shredded but often no identifiable site, 4 identifiable but flat, 5 perfect or nearly. Pictures were taken prior to evaluation for documentation. Two experimenters scored the nest blind and independently of each other. Neophobia was measured using the corner index recorded in standard transparent home-cages. In a 30 s period, the number of visited corners, as well as the rearings in the corners were counted as described^5^. Anxiety behavior was examined in the dark/light box (0,50 m × 0,50 m) divided into same size dark and light sides. The mouse was placed in the dark side of the box for 10 sec acclimatization. A door separating the two compartments was opened and the amount of time that the mouse took to emerge fully (all four paws) into the open area, total time in the light area, and number of transitions were measured for 5 min. Hippocampus-dependent spatial memory abilities were examined in the hidden-platform version of the water maze as described^59^. Time required to locate the hidden platform (escape latency), distance traveled (path length), and swimming speed were recorded using the Ethovision video tracking system (Noldus Information Technology, Wageningen, The Netherlands) as described^56,60^. Swim paths for each mouse in each trial were plotted and categorized into eight mutually exclusive categories: thigmotaxis, chaining, random search, scanning, directed search, focal incorrect, focal search and direct swimming to the platform, of which the latter three are considered true spatial strategies. Two days following the acquisition phase, a 100 sec probe trial, in which the platform was removed, was performed. We visualized swimming paths using a custom-made MATLAB protocol. Single-trial passive avoidance learning was examined in a grid-floor apparatus (MED Associates Inc., St. Albans, Vermont, USA) as described^40,60^. Briefly, mice were dark adapted for 30 min, and then placed in the small illuminated compartment. The sliding door to the dark compartment was opened after 5 sec, and the entry latency was recorded. When mice entered the dark compartment a foot shock (0.3 mA, 1 sec) was delivered. Retention was tested 24 h later without delivering the shock.

### Biochemical analysis

For biochemical analysis, half hippocampi were lysed in cold-lysis buffer (62.5 mM Tris hydrochloride, pH 6.8, 10% glycerol, 5% β-mercaptoethanol, 2.3% sodium dodecyl sulfate [SDS], 5 mM NaF, 100 µM Na_3_VO_4_, 1 mM EDTA, 1 mM EGTA) containing protease and phosphatase inhibitors (Roche España, Barcelona, Spain) and boiled at 100 °C^61^. Protein content was quantified with the Coomassie (Bradford) protein assay kit (Thermo Fisher Scientific, USA), resolved on SDS-polyacrylamide gel electrophoresis and detected by Western blotting using the following antibodies: rabbit anti-tau (Tau17025; 1:5000), mouse anti-phosphorylated Thr231 (AT180; 1:200), Ser202 (CP13; 1:250), and Ser396/404 (PHF-1; 1:250), abnormal tau conformation (aa 5–15 and 312–322; MC1), total tau TG5 (Tau 220–242; 1:500), and phosphorylated Ser9 GSK3β (1:1000; Cell Signaling, Danvers, Massachusetts) and anti-GSK3β (1:2500; BD Biosciences, San Jose, CA, USA), GFAP (1:250; Agilent, Santa Clara, CA, USA) and GAPDH (1:100000; Thermo Fisher Scientific). Bands were detected with enhanced chemiluminescent reagent in a ChemiDoc MP System (Bio-Rad) and quantified in a linear range using the ImageLab 5.2.1 software. Original blot images are shown in Supplementary Information.

### Immunohistochemical analysis

For immunohistochemical analysis, vehicle- and Se-treated WT and 3xTg-AD mice (n = 6–7/group) were perfused with PBS followed by 4% paraformaldehyde. Left brain hemispheres were dehydrated in ethanol before paraffin embedding. Coronal brain sections (5 μm) were deparaffinized in xylene, rehydrated and incubated with 3% hydrogen peroxide in methanol. For Aβ staining, sections were treated with 60% formic acid (6 min), a protocol that allows specific labeling of Aβ over full-length APP in APP transgenic mice as described^6^. For tau and GFAP staining, sections were pretreated with citrate buffer and microwave heated (10 min) to allow antigen retrieval. Sections were washed in TBS, incubated overnight with antibodies against human Aβ1-16 (6E10; 1:1,000; Covance), hyperphosphorylated tau (PHF1, 1:50; CP13, 1:50), conformational aggregated tau (MC1, 1:20) or GFAP (1:200), and processed for avidin-biotin immunoperoxidase staining using the Vectastain Elite ABC kit (Vector Laboratories, Burlingame, CA)^6^. Sections corresponding to bregma −2, −2.5, −3, −4 and −5 mm (n = 5/mouse) were stained and imaged (10×) using a Nikon Eclipse 90i microscope (Nikon Instruments Europe, Amsterdam, The Netherlands). We quantified GFAP-stained area  and the number of dense, sparse and total amyloid plaques, intraneuronal Aβ-positive neurons and phosphorylated tau-positive neurons in multiple regions/fields (680 × 840 μm) of distinct sections (≥4) of 3xTg-AD Veh (n = 6) and 3xTg-AD Se (n = 7) mice using ImageJ.

### Statistical analysis

Behavioral comparisons were conducted with one-way analysis of variance (ANOVA) followed by Bonferroni *post-hoc* test. Morris water maze results were analyzed with two-way ANOVA for repeated measures (RM), followed by Bonferroni *post-hoc* test by using SigmaStat software (Systat Software, San Jose, CA). Statistical analyses of pathological and biochemical studies were performed using t-test (immunohistochemistry) or two-way ANOVA followed by Bonferroni *post-hoc* test for multiple comparisons (biochemical studies) by using Prism software (GraphPad, La Jolla, CA). *P < 0.05, **P < 0.01, ***P < 0.0001.

### Ethical experimental statement

This study was performed in accordance with the experimental European Union guidelines and regulations (2010/63/EU). Experimental protocols and experiments involving vertebrate animals were conducted in accordance with the ethical protocol approved by the Animal and Human Ethical Committees of the Katholieke Universiteit Leuven and Universitat Autònoma de Barcelona (protocol number: CEEAH 2896) and the local Governmental Ethical Committee Generalitat de Catalunya (protocol number: DMAH 8787).

### Data availability statement

All data generated during this study are included in this published article or are available from the corresponding author upon reasonable request.

## Electronic supplementary material

Supplementary Information
