# Supplementary material for: Reversal of memory and neuropsychiatric symptoms and reduced tau pathology by selenium in 3xTg-AD mice
Source: Sci Rep. 2018 Apr 24;8:6431. doi: 10.1038/s41598-018-24741-0 (PMC5915484; doi:10.1038/s41598-018-24741-0)

Supplementary Information

Reversal of memory and neuropsychiatric symptoms and reduced tau pathology by selenium in 3xTg-AD mice

Ann Van der Jeugd, Arnaldo Parra-Damas, Raquel Baeta-Corral, Carlos M. Soto-Faguás, Tariq Ahmed, Frank M. LaFerla, Lydia Giménez-Llort, Rudi D'Hooge, Carlos A. Saura*

Original blots shown in Figures 4B and 5B.

Images correspond to original scans of independent blot membranes (upper and lower images within the same gel) containing multiple hippocampal samples (n=4-5/group) blotted with the specific antibodies against the indicated proteins.

The black discontinuos square lines indicate the part of the original image cropped for composition of the original Figures 4B and 5B. Molecular weight markers are indicated on the left.

CP13

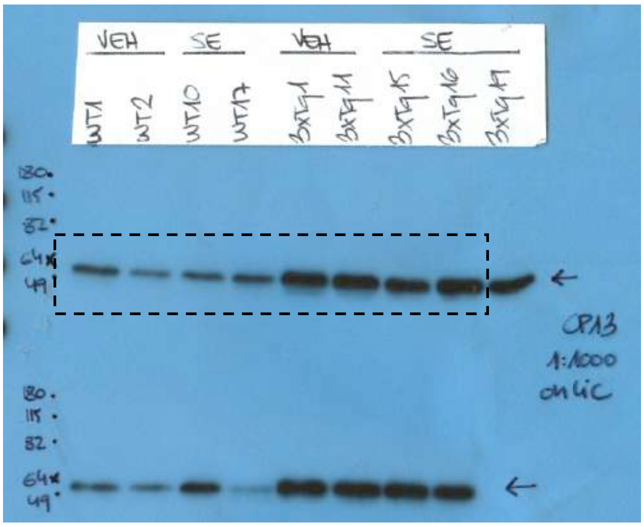

AT180

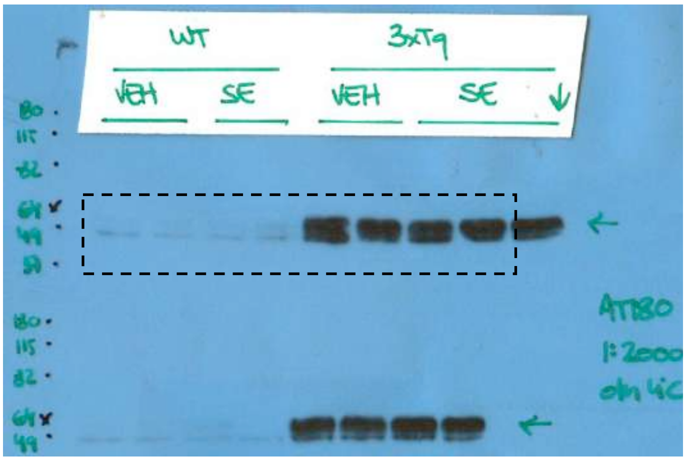

PHF1

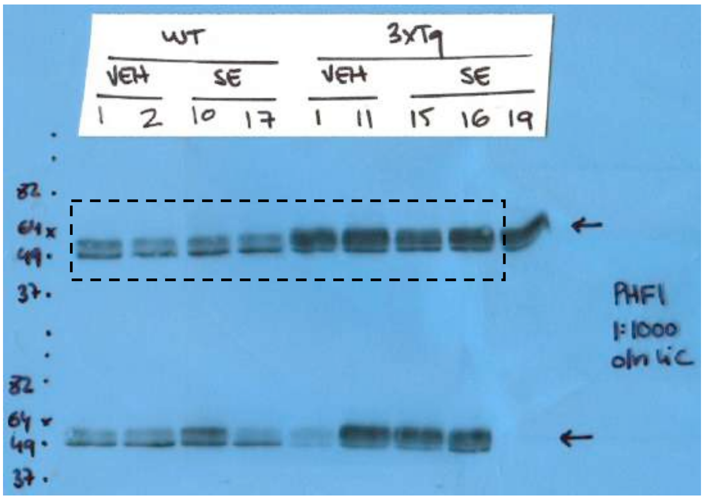

TG5

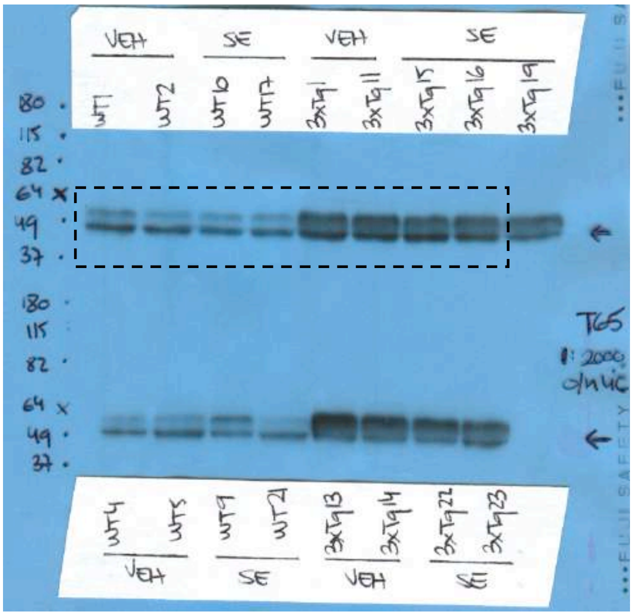

Tau17025

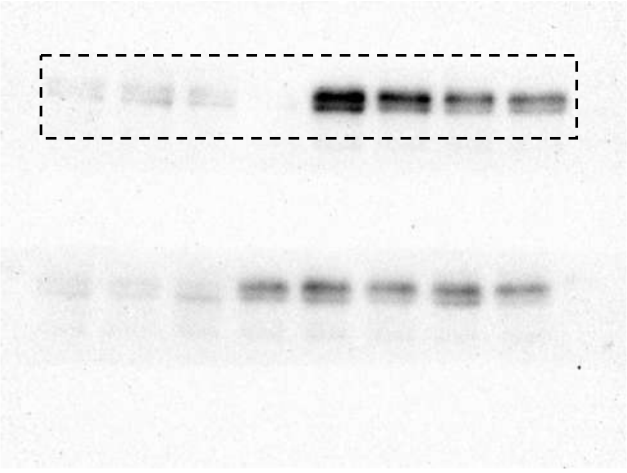

GAPDH

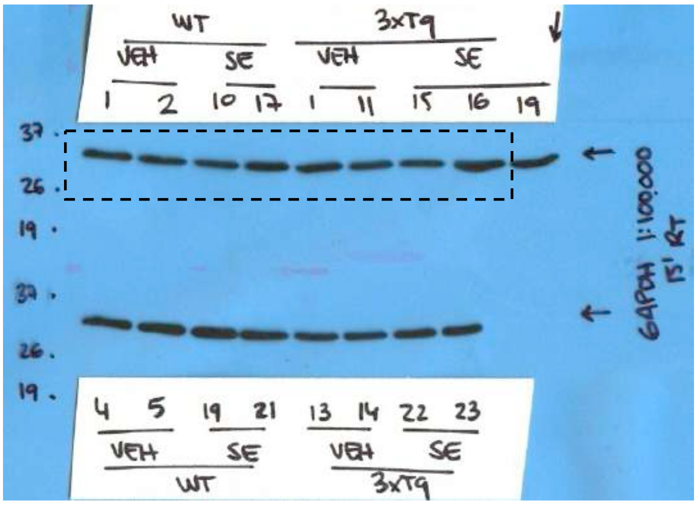

GSK3β Ser9

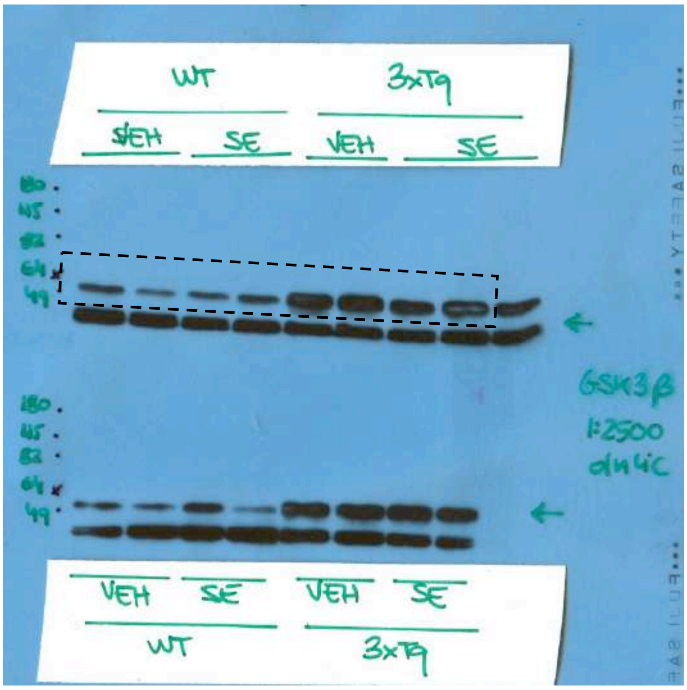

GSK3β

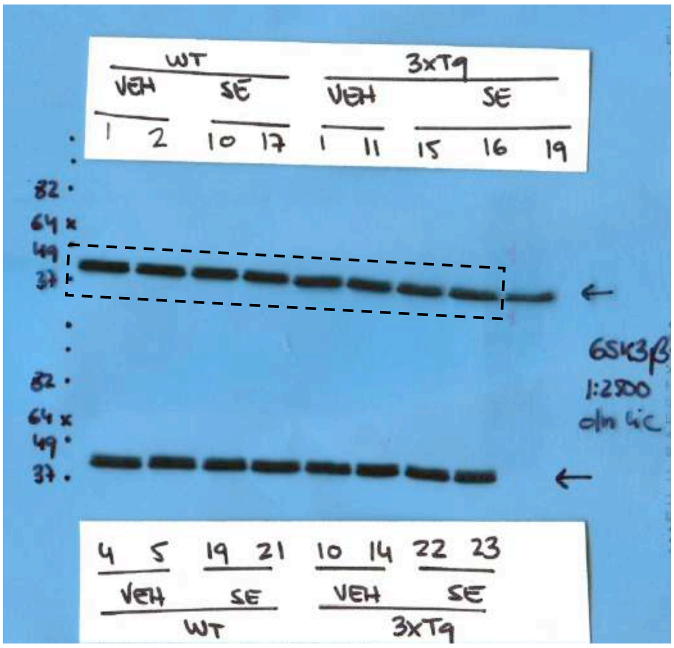

GAPDH

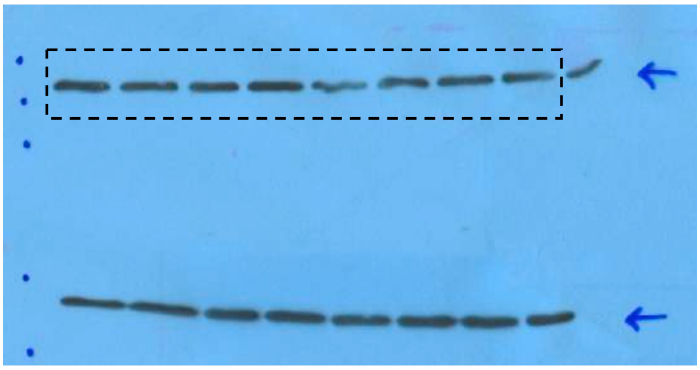

Figure 5B

GFAP **GAPDH**

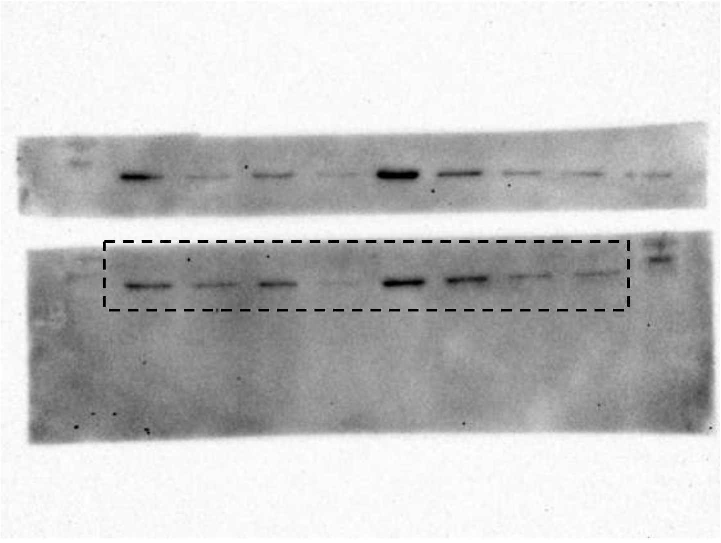

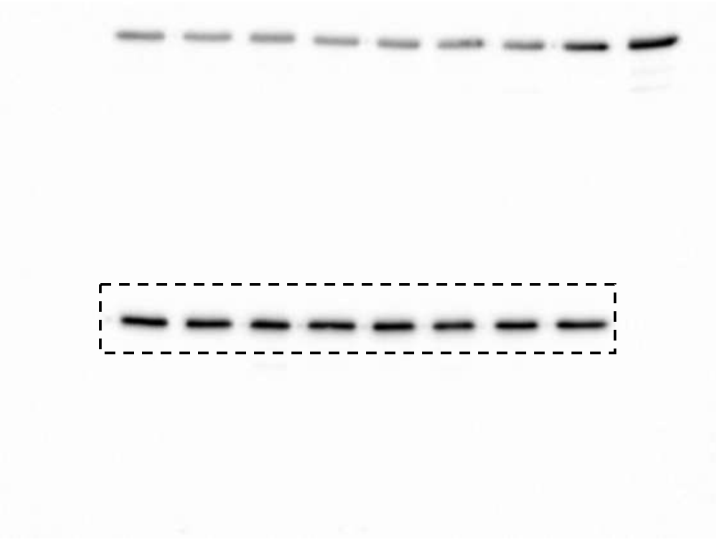

Supplement: Supplementary file 1 — Supplementary Information [file 41598_2018_24741_MOESM1_ESM.pdf]
